# A High-Throughput Assay for Screening of Natural Products that Enhanced Tumoricidal Activity of NK Cells

**DOI:** 10.1186/s12575-015-0026-6

**Published:** 2015-10-28

**Authors:** Chenyuan Gong, Zhongya Ni, Chao Yao, Xiaowen Zhu, Lulu Ni, Lixin Wang, Shiguo Zhu

**Affiliations:** Laboratory of Integrative Medicine, School of Basic Medical Sciences, Shanghai University of Traditional Chinese Medicine, 1200 Cai Lun Road, Shanghai, 201203 P.R. China

**Keywords:** Natural products, NK cells, High-throughput screening, IFN-γ, Andrographolide

## Abstract

**Background:**

Recently, immunotherapy has shown a lot of promise in cancer treatment and different immune cell types are involved in this endeavor. Among different immune cell populations, NK cells are also an important component in unleashing the therapeutic activity of immune cells. Therefore, in order to enhance the tumoricidal activity of NK cells, identification of new small-molecule natural products is important. Despite the availability of different screening methods for identification of natural products, a simple, economic and high-throughput method is lacking. Hence, in this study, we have developed a high-throughput assay for screening and indentifying natural products that can enhance NK cell-mediated killing of cancer cells.

**Results:**

We expanded human NK cell population from human peripheral blood mononuclear cells (PBMCs) by culturing these PBMCs with membrane-bound IL-21 and CD137L engineered K562 cells. Next, expanded NK cells were co-cultured with non-small cell lung cancer (NSCLC) cells with or without natural products and after 24 h of co-culturing, harvested supernatants were analyzed for IFN-γ secretions by ELISA method. We screened 502 natural products and identified that 28 candidates has the potential to induce IFN-γ secretion by NK cells to varying degrees. Among the 28 natural product candidates, we further confirmed and analyzed the potential of one molecule, andrographolide. It actually increased IFN-γ secretion by NK cells and enhanced NK cell-mediated killing of NSCLC cells.

**Conclusions:**

Our results demonstrated that this IFN-γ based high-throughput assay for screening of natural products for NK cell tumoricidal activity is a simple, economic and reliable method.

## Background

Natural killer (NK) cells are derived from hematopoietic progenitor cells and are abundantly present in bone marrow, various organs and even in secondary lymphoid tissues [1]. They constitute nearly 10–15 % of PBMCs and are usually defined by expression of CD56 and CD16 proteins, but lacks CD3 [2, 3]. NK cells are a critical component of the innate immune response against tumor cells and infections [4–6]. The tumoricidal activity of NK cells is mediated by release of, a) various cytokines such as IFN-γ and TNF-α, b) exocytosis of granzymes (Perforin) containing lytic granules [7], and c) expression of some death receptor ligands [8]. NK cells recognition and destruction of tumor cells does not involve prior sensitization in an MHC-I-unrestricted manner [9]. Thus, NK cells immunotherapy have shown a great potential in most of the hematopoietic malignancies [10].

There have been innumerable attempts over years to use NK cell immunotherapy for the treatment of solid cancers without much success. NK cells-mediated targeting of solid tumor is usually not efficient despite of the tumor cells expressing high amounts of activating and low levels of inhibitory ligands [11]. This is attributed to the creation of an immunosuppressive environment by cancer cells to evade NK cell immune surveillance, through secretion of factors like TGF-β [12, 13]. Thus, it leads to poor prognosis [14]. The escape of tumor cells from immunosuppressive cells like NK cells, leads to the failure of the immunotherapy especially for solid tumors in the clinical settings. Hence, the development of new drugs with ability to block the immunosuppressive environment is required to efficiently block the escaping of tumor cells from immunosuppressive cell and will help in restoring NK cell-mediated anti-tumor response [15].

Small-molecules isolated from natural products are a major source for the development of new drugs. So far, almost 50 % of all the new drugs were either natural products or their analogs, including those that modulate immune cell functions [16]. Natural products that enhance NK cell antitumor activity can block the escaping of tumor cells from immune response and restore NK cell mediated antitumor activity. However, lack of simple and economic approach for large scale screening of the natural products has been a big impediment and thus warrants a development of a novel high-throughput assay for screening of novel and efficient small molecules from a pool of thousands of natural products.

IFN-γ is a major cytokine secreted by activated NK cells and exerts immune response against cancer and virus-infected cells by inducing tumor apoptosis [17, 18]. IFN-γ has is a potent indicator of NK cell activity. Bellucci R. et al. showed that silencing of 83 genes in tumor cells resulted in enhanced IFN-γ release from NKL cells and thus promoted apoptosis [19]. Similarly, Lee SB. et al. also assessed NK cell activity as a function of IFN-γ secretion in whole blood samples [20]. Therefore, in this study, we also developed an IFN-γ based high-throughput assay screen for assessing the effect of small-molecule natural products on the tumoricidal activity of NK cells. After screening 502 natural products, we identified 28 candidates with a potential to enhance NK cell activity and further confirmed that andrographolide, one among the 28 natural products, actually induced IFN-γ secretion by NK cells, and enhanced NK cell mediated killing of cancer cells.

## Results

### Human NK Cell Expansion

**Fig. 1:**
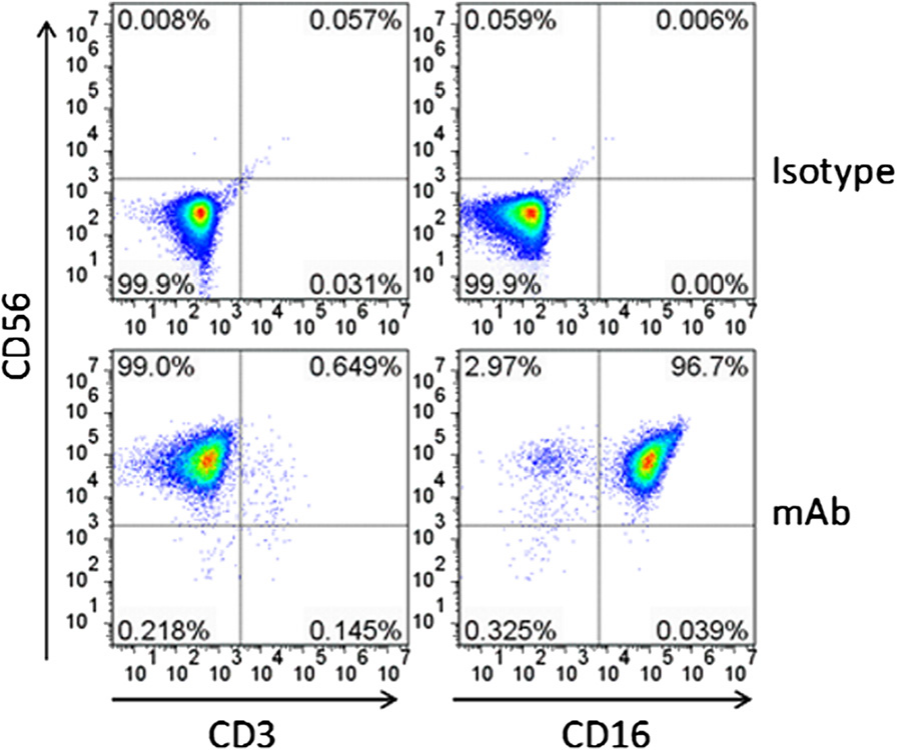
Expansion of human NK cells. NK cells were expanded as described in Methods section, and were stained with CD56, CD16 and CD3 antibodies for flow cytometry analysis. The data set presented here is a representative of three independent experiments

**Fig. 2:**
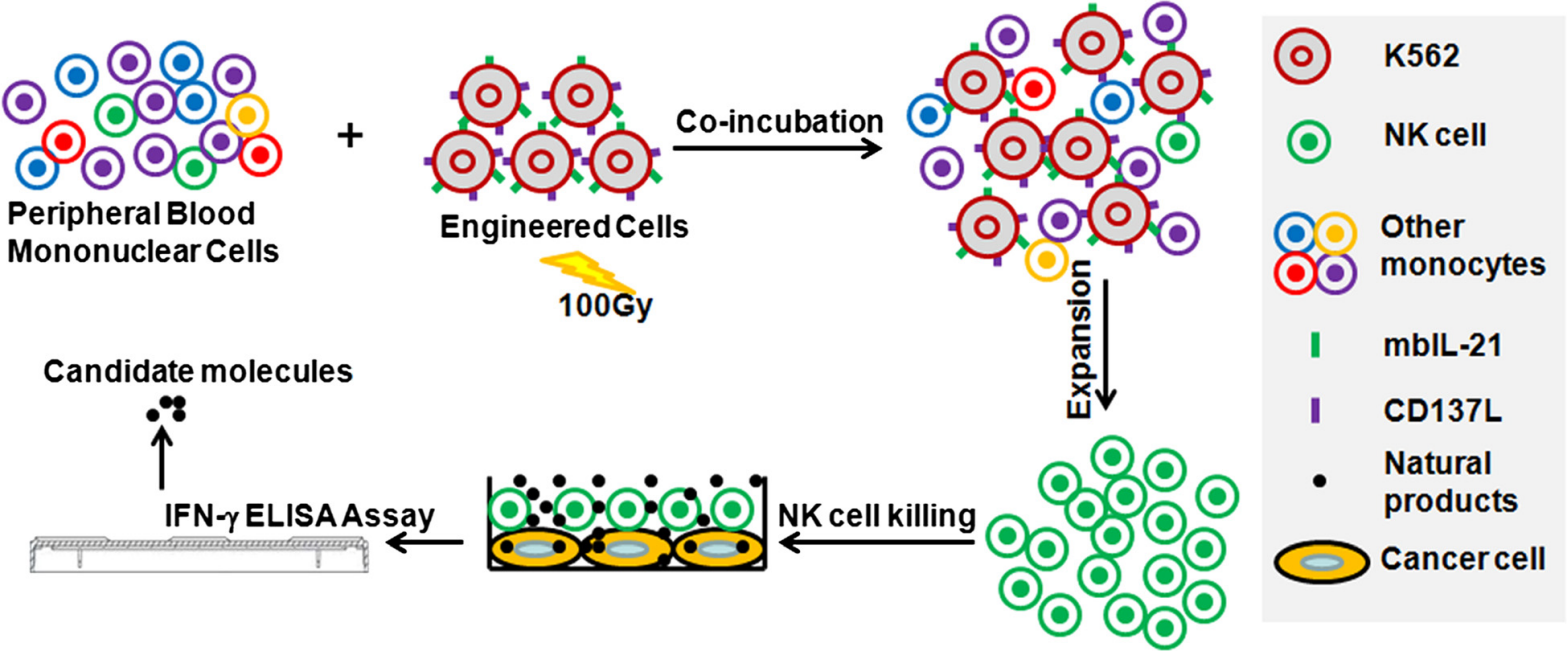
The schematic presentation of IFN-γ based high-throughput assay for screening of natural products. To identify potent natural products, human NK cells were first expanded, and then co-incubated with A549 cells in 96-well plates with or without natural products. After 24 h of co-culturing, supernatants were harvested and IFN-γ levels were measured by ELISA assay

In clinical application, there is always a requirement of high number of NK cells and therefore, we first expanded the NK cell population by an efficient system developed by us previously for NK cell expansion [21]. The CD137L and membrane-bound IL-21 engineered K562 were irradiated and then co-incubated with PBMCs for 2 weeks. Results showed that about 99 % of CD3^−^CD56^+^ cells were NK cells after 2-week of expansion. Among these NK cells, majority of them were CD56^+^CD16^+^ cells (>95 %) (Fig. 1). These results were consistent with our previous result, and indicated that these expanded NK cells were of high purity, and were subsequently used for screening of natural products (Fig. 2).

### Optimization of Co-culturing Conditions for Maximum NK Cell Tumoricidal Response

**Fig. 3:**
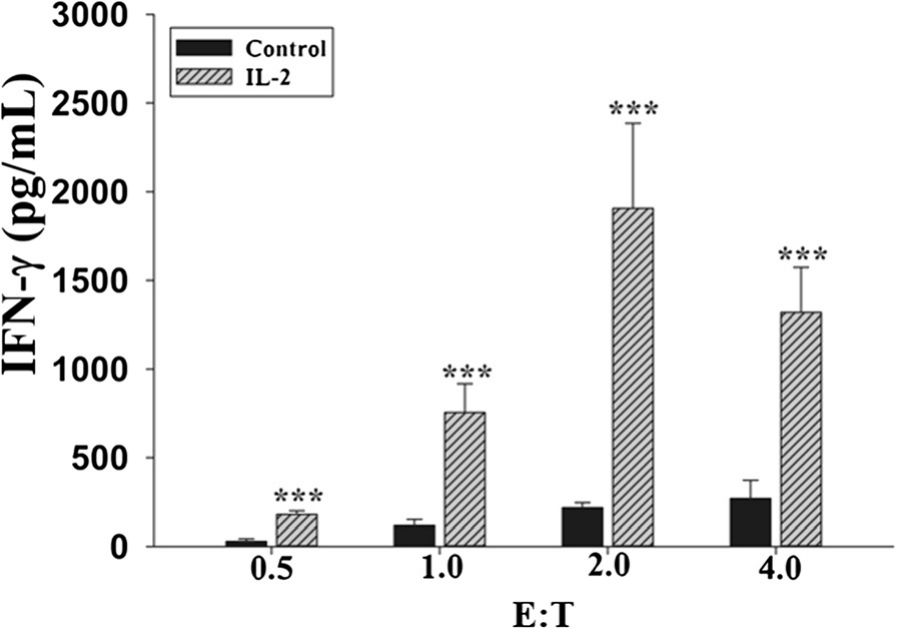
Optimization of NK and target (A549) cells culturing conditions. NK cells were co-cultured with A549 cells at different E:T ratios in 96-well plates for 24 h, and then IFN-γ secretion in supernatant was detected by ELISA assay. The data shown here is a representative of three independent experiments. ***, *P*<0.001

NK cells release IFN-γ after recognizing and binding to the target cells. The level of IFN-γ production is directly proportional with NK cell tumoricidal activity. Therefore, we first optimized the E:T ratio of NK cells (effector) and A549 tumor cells (target) for inducing efficient tumoricidal activity. Herein, we used IL-2 as a positive activator/stimulator to induce NK cells cytotoxic activity by co-culturing them with target cells. We mixed lung adenocarcinoma, A549 cells (5×10^3^) per well together with different ratios of NK cells (NK cells: A549 cells=0.5:1, 1:1, 2:1, 4:1) in 96-well plate either in the presence or absence of 100U/mL of IL-2 for 24 h. Subsequently, IFN-γ secretion was measured in the supernatants. Results showed that secreted IFN-γ was enhanced with the increasing ratio of NK cells to target cells after IL-2 stimulation and peaked at 2:1 ratio (Fig. 3). These results demonstrated that 0.5×10^4^ of A549 cells mixed with 1×10^4^ of NK cells could elicit maximal IFN-γ secretion after IL-2 stimulation, thus, indicating that this E:T ratio is optimum for screening of natural products.

### Screening of Natural Products for Induction of NK Cell Tumoricidal Activity

**Fig. 4:**
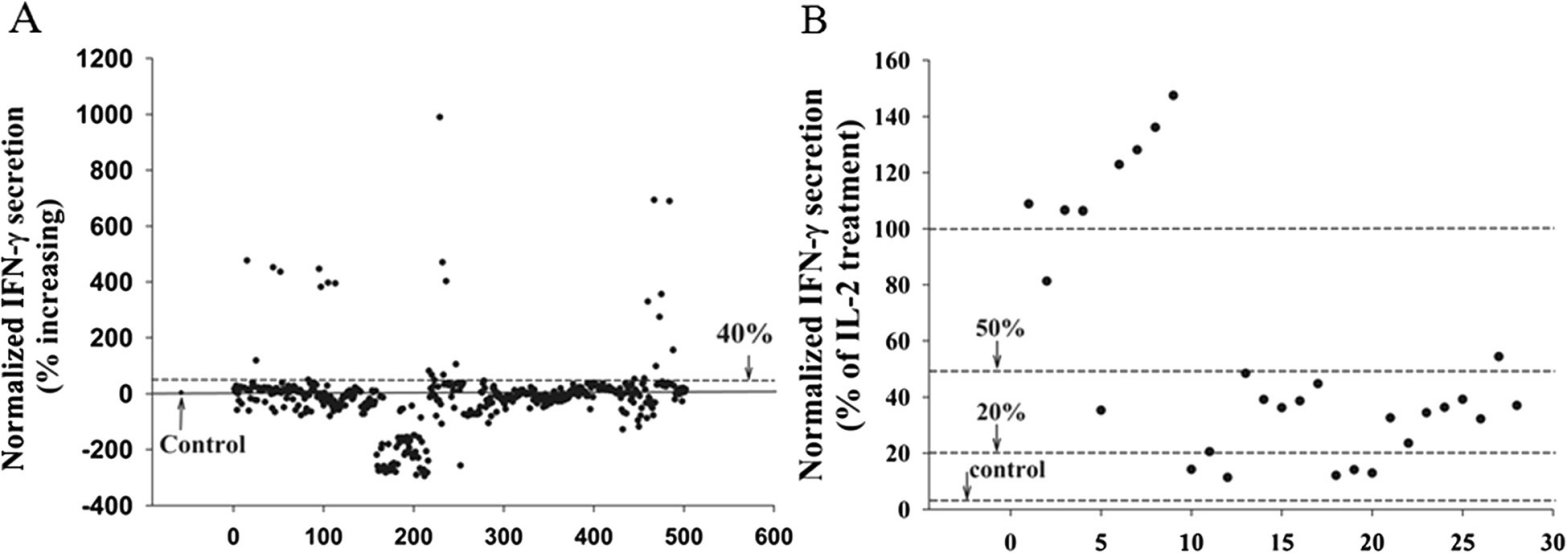
Screening of natural products. Expanded NK cells were mixed with A549 cells in 96-well plates and natural products were added to a final concentration of 10 μM. Panel **a**, represents percentage increase of IFN-γ after normalization to DMSO control. Panel **b**, represents IFN-γ percentage ratio after normalization to IL-2 treatment

To determine the efficiency of 502 natural products to induce NK cell tumoricidal activity, we plated 1×10^4^ of expanded NK cells from 3 donors and 5×10^3^ of A549 cells per well in 96-well plates. Thereafter, these co-cultured cells were treated with each of the natural products at a final concentration of 10 μM. At parallel, the cells were also treated with equal volume of DMSO as negative control and 100 U/mL of IL-2 as a positive control. We arbitrarily used ≥ 40 % increase in IFN-γ secretion, in comparison to the negative control, as efficient criteria. Based on this criterion, we identified that 28 natural products induced an efficient increase in IFN-γ secretion (Fig. 4a). Comparison of IFN-γ secretion induced by 28 natural products and corresponding IL-2 stimulations, we found that IFN-γ secretion by 5 natural products were < 20 % of the levels induced by IL-2, whereas 14 natural products showed 20 %–50 % of the IFN-γ secretion in comparison to IL-2 induction (Fig. 4b). Interestingly, 2 natural products displayed 50 %–100 % of IFN-γ secretion and 7 natural products had >100 % secretion in comparison to IL-2 induced IFN-γ secretion. Among these 7 natural products, 6 of them were protein kinase C activators and another natural product was unknown.

### Effect of Andrographolide on Viability of NK and NSCLC Cells

**Fig. 5:**
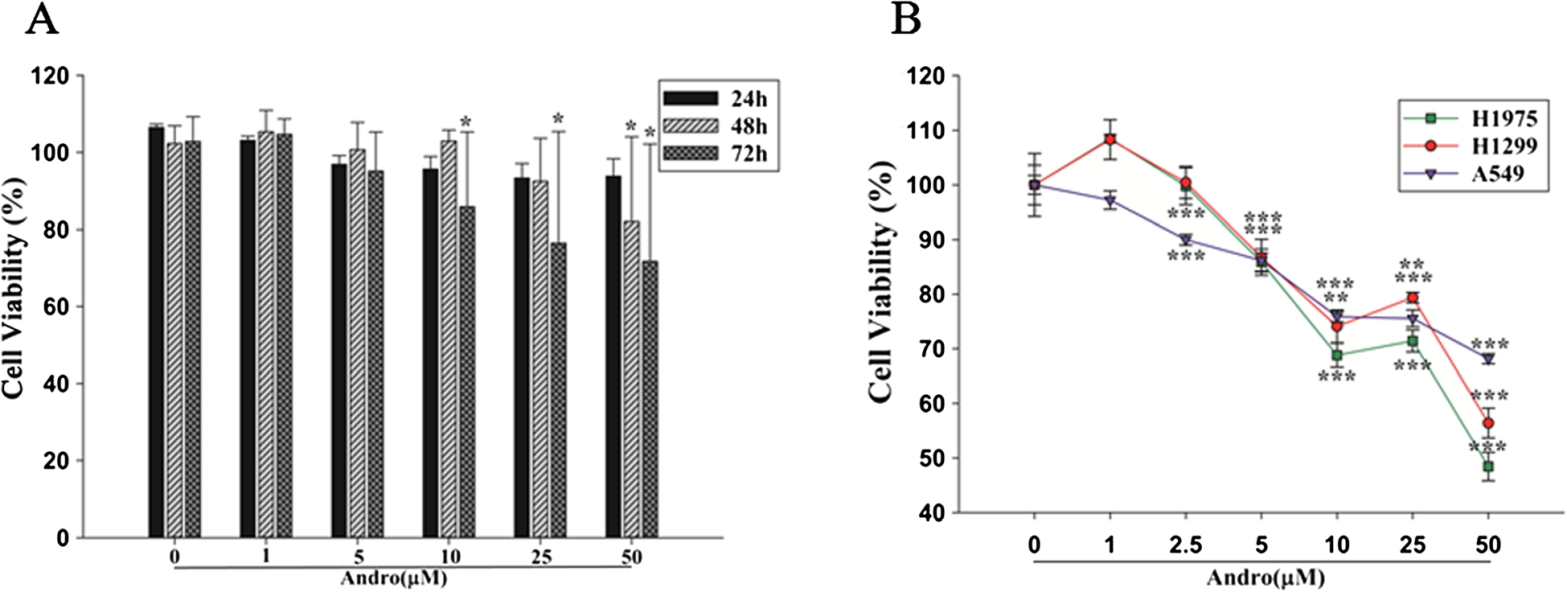
Analysis of andrographolide effects on cell viability of NK and NSCLC cells. Expanded NK cells and NSCLC cells were treated with different concentrations of andrographolide (Andro) for 24, 48 and 72 h, respectively. Panel **a**, represents NK cell viability as determined by CCK8 assay. Panel **b**, represents NSCLC cell viability as assessed by MTT assay. Three independent experiments were performed. Data are expressed as means ± S.E.M. *, *P*<0.05; **, *P*<0.01; ***, *P*<0.001

Andrographolide, one among the 28 natural products, has been found to suppress vascular angiogenesis and tumor growth in our previous studies [22]. This led us to further analyze this natural product and we investigated its effects on NK cells and NSCLC cells viability. Expanded NK cells and different NSCLC cell lines (A549, H1299 and H1975) were treated with different concentrations of andrographolide (0, 1, 5, 10, 25 and 50 μM). Results showed that all selected doses of andrographolide did not impair NK cells viability at 24 h time point. But 50 μM concentration of andrographolide had 20 % inhibition (*p*<0.05) at 48 h; and 10, 25 and 50 μM of andrographolide had 15 %, 20 % and 30 % inhibition (*p*<0.05) at 72 h (Fig. 5a). On the other hand, andrographolide above 2.5 μM significantly (*p*<0.001) suppressed the cell viability of all NSCLC cell lines at all time points (Fig. 5b). These results thus indicate that andrographolide has low toxicity towards expanded NK cells, but has higher toxicity against NSCLC cells.

### Role of Andrographolide in Inducing NK Cell Mediated IFN-γ Secretion and Killing

**Fig. 6:**
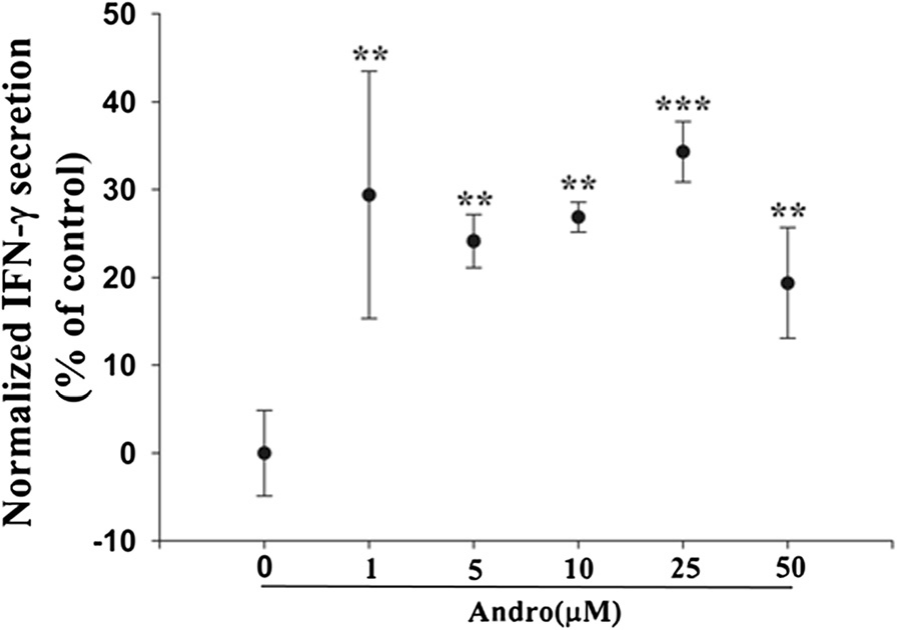
Andrographolide increased IFN-γ secretion by NK cells. Expanded NK cells and A549 cells were co-cultured with various concentrations (1 μM to 50 μM) of andrographolide. After 24 h, IFN-γ release in supernatant was measured by ELISA method

**Fig. 7:**
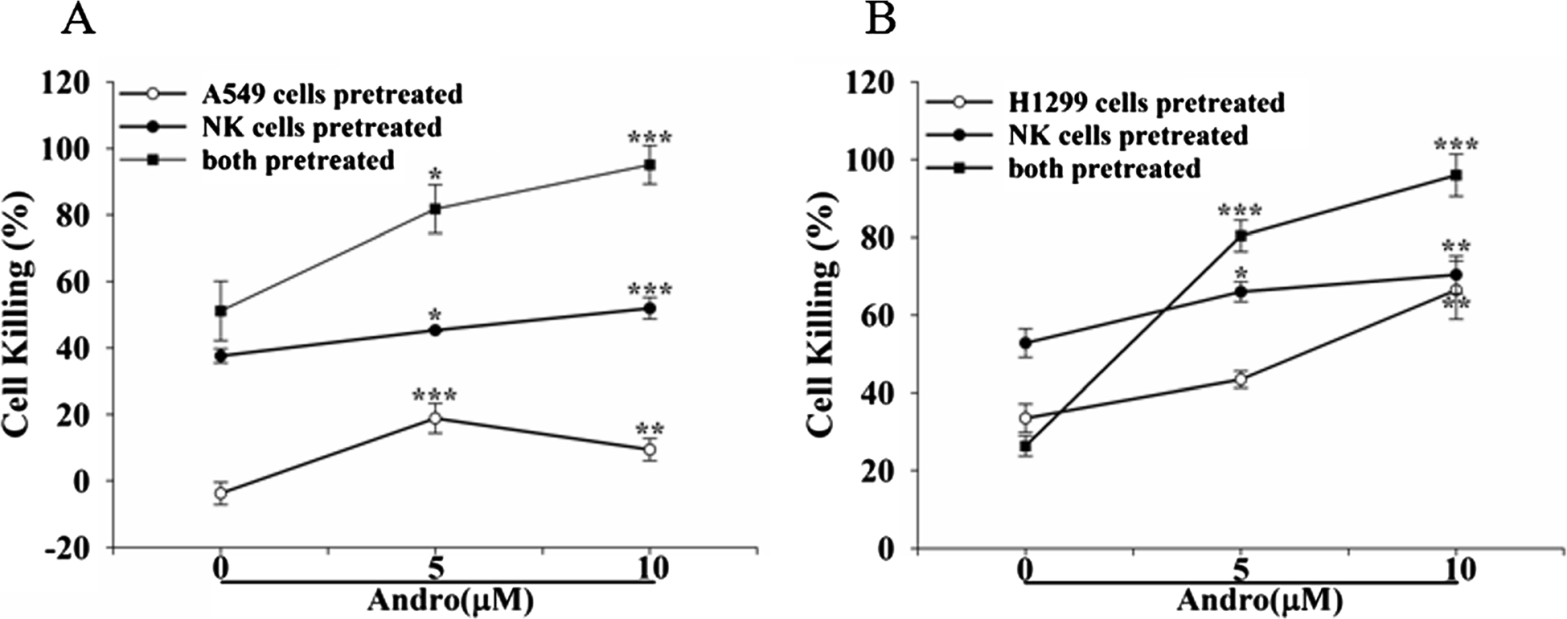
Andrographolide enhanced NK cell tumoricidal activity. NSCLC cells or NK cells were pretreated for 24 h with andrographolide, 5 and 10 μM concentrations, respectively. Later NK cells cytotoxicity was evaluated by calcein release assay with a 5:1 ratio of effectors to target cells. Panel **a**, A549 cells; Panel **b**, H1299 cells. The data shown here are representative of three independent experiments. Data are expressed as means ± S.E.M. *, *P*<0.05; **, *P*<0.01; ***, *P*<0.001

Since low doses of andrographolide did not inhibit the cells viability of expanded NK cells but had significant suppression in NSCLC cells, we next focused on determining its effect on NK cell tumoricidal activity. We first incubated NK cells and A549 cells together with different concentrations of andrographolide for 24 h, and then detected IFN-γ release by NK cells. The results showed that andrographolide increased IFN-γ release, which was not clearly dose-dependent (Fig. 6). Next, we determined if andrographolide enhanced the NK cell-mediated lysis of NSCLC cells by calcein release assay. Here we pretreated the A549 and H1299 NSCLC cells with 5 and 10 μM of andrographolide for 24 h, respectively. Later these andrographolide pretreated NSCLC cells were incubated with untreated NK cells and we observed that NK cells significantly enhanced the lysis of andrographolide treated A549 and H1299 cells. Further, we pretreated the NK cells with andrographolide and then incubated them with untreated NSCLC cells, and again observed a significant enhancement of NK cell mediated lysis of A549 cells and H1299 cells. Finally, we pretreated both NSCLC cells and NK cells, respectively; and then incubated them together, and observed this time also a significant enhancement of NK cell killing (Fig. 7a, b). Taken together, these results indicated that andrographolide treatment has the potential to enhance NK cell tumoricidal activity.

## Discussion

In this study, we developed an IFN-γ-based high-throughput screening assay to screen natural products which might enhance NK cell mediated lysis of cancer cells. By measuring the IFN-γ release by NK cells, we totally screened 502 natural products and identified that 28 natural products could increase IFN-γ release. Andrographolide, one among 28 natural products, displayed low toxicity in expanded NK cells but had higher toxicity against different NSCLC cells. But, andrographolide not only induced IFN-γ release but also enhanced NK cell mediated killing of tumor cells. These results thus established that the IFN-γ-based screening assay is a viable option for screening and identifying natural products that has the potential to enhance NK cell mediated killing of cancer cells.

Previously published studies have shown that natural products are a good source of compounds that can improve NK cell anti-tumor response by enhancing NK cell mediated recognition and killing of cancer cells [23–27]. However, it was hard to find efficient candidates from millions of natural products [28]. Numbers of different methods have been developed so far to detect NK cell cytotoxicity. For example, Chromium 51 release assay is the standard method to determine NK cells cytolytic activity. In addition, CD107α degranulation assay and several flow cytometric methods were also alternatively used to determine NK cell cytotoxicity [29]. However each of these assays had certain limitations, for example, chromium 51 release assay were high-cost, involved hazardous radioactivity and generated radioactive waste [30], whereas CD107α degranulation assay and other flow cytometric methods were time consuming and involved high cost, and more importantly, all these methods were not suitable for high-throughput analysis [31]. Furthermore, these methods only focused on the final NK cell killing activity on cancer cells, and totally ignored, the inhibitory influence of cancer microenvironment, such as IDO (Indoleamine-2,3-dioxygenase), PGE2 (Prostaglandin E2), and other factors secreted by cancer cells against NK cells [32]. Therefore, the development of a high-throughput screening assay that could mimic the cancer microenvironment was necessary for identification of natural products that has the potential to modulate NK cell activity.

IFN-γ is a good indicator of NK cell cytotoxicity and has been used to determine the NK cells anti-tumor activity in whole blood [20]. In this study, we had developed an IFN-γ-based assay to determine NK cell killing activity in an *in vitro* NK cell-cancer cell interacting microenvironment. To get an optimal IFN-γ response, we optimized E/T ratio and found the best E/T ratio was 2. This E/T ratio may change in different cancer model and was not equal to the best killing effect. In fact, more NK cells may exert much higher killing efficacy, but the IFN-γ response may be not optimal. As shown in Fig. 3, INF-γ product become lower at E/T ratio 4 compared to E/T ratio 2. We analyzed 502 natural products for induction of IFN-γ release by NK cells and identified that 28 natural products induced 40 % increase compared to no treatment. Among these natural products, 7 induced higher IFN-γ secretions than IL-2 stimulation and out of these 7 natural products, 6 were protein kinase C activators. This result was consistent with previous published reports that protein kinase C activation could increase IFN-γ secretion [33], and suggested that protein kinase C may be a good target for natural products-induced NK cell activation [34, 35].

Andrographolide, one among the 28 natural products had been demonstrated to have anti-inflammatory, anticancer and angiogenesis activities both *in vitro* and *in vivo* [22, 36]. In this study, andrographolide was shown to have a low toxicity towards expanded NK cells, but displayed higher toxicity against NSCLC cells, which was consistent with its anti-tumor effects. In addition, andrographolide really enhanced IFN-γ release by NK cells and also NK cell-mediated killing of NSCLC cells, and this enhancement was dose-independent. Dose-independent effect is a common phenomenon. Fox example, small-molecule antagonist BIO-1211 (Very Late Antigen-4 (VLA4) blocker) results in reduced cytokines expression, leukocyte trafficking, and inhibition of inflammatory responses in EAE in a dose-independent manner [37]. Of course, the mechanism is complicated and additional experiments are needed to further define. Taken together, our results suggested that andrographolide might have a potent application in NK cell immunotherapy, and that the IFN-γ based high-throughput screening assay can be a reliable method.

## Conclusions

In this study, we developed a simple and economic, IFN-γ based high-throughput screening assay to identify natural products that could enhance the NK cells tumoricidal activity. Furthermore, with the application of this assay, we identified 28 natural product candidates. This high-throughput screening assay might have valuable application in NK cell-based drug discovery, and the 28 natural product candidates can have potent application in modulation of NK cell function and immunotherapy.

## Methods

### Reagents

APC anti-human CD56, FITC anti-human CD3, PE anti-human CD16, murine isotype controls (IgG1κ-PE, IgG1κ-FITC, IgG2a –APC) and human IFN-γ ELISA MAX™ Deluxe kit were purchased from BioLegend Inc. (San Diego, CA). The recombinant human IL-2 protein was obtained from PeproTech (Rehovot, Israel). Calcein-AM was purchased from Sigma-Aldrich (St, Louis, MO). CCK8 kit was purchased from YESAN (Shanghai, China).

### NK Cell Expansion

Human peripheral blood mononuclear cells (PBMC) were obtained from the Shanghai Blood Center under a research protocol approved by the Department of Shanghai Blood Administration. PBMCs were either freshly used or frozen in 10 % DMSO containing fetal bovine serum (FBS, Gibco). Frozen PBMCs were thawed, one day prior to their cultivation in RPMI1640 medium supplemented with 10 % of fetal calf serum, 1 % of penicillin-streptomycin, 2 mM of L-Glutamine and 200 U/ml of IL-2 in 5 % CO2 at 37 °C. NK cells were expanded as described previously [21]. Briefly, fresh or frozen PBMCs were co-incubated with mbIL-21-CD137L-K562 cells which were pretreated by 100 Gy irradiation for 2 weeks in RPMI1640 complete medium in 5 % CO2 at 37 °C.

### Flow Cytometry Analysis

Cells were incubated with CD56, CD16 and CD3 antibodies conjugated with different fluorophores for 30 min at 4 °C in dark, followed by washing, and resuspension in PBS containing 1 % FBS. Data were acquired using a BD Accuri C6 (BD Biosciences) and analyzed using the FlowJo software (Ashland, OR).

### Screening Assay

Total of 502 natural products were obtained from China National Compound Resource Center (NCRC). The NK cells (1×10^4^) from 3 donors were mixed with A549 lung adenocarcinoma cells (5×10^3^) per well in 96-well plates. These co-cultured cells were treated with natural products to a final concentration of 10 μM. Equal volume of DMSO was added as negative control and 100 U/mL of IL-2 was added as positive stimulator. After 24 h of incubation, supernatants were harvested and IFN-γ was measured by Human IFN-γ ELISA Kit following the manufacturer’s instructions. Briefly, IFN-γ (capture) antibody was incubated in 96-well plates at 4 °C overnight. The plates were then washed 3 times with PBS containing 0.1 % Tween-20, incubated with blocking buffer for 1 h, and then washed 3 times again with PBS containing 0.1 % Tween-20. Supernatant were added to each well and incubated for 2 h, followed by washing of plates again 3 times with PBS containing 0.1 % Tween-20. Next, detection antibody was added and plates were incubated for 1 h. At last, HRP reagent was added for colorimetric detection of the signal and plates were read at 450 nm using Synergy 2 Multi-Mode Microplate Reader (BioTek Instruments, Inc., Winooski, VT).

### Cell Viability Assay

NK cells were seeded into 96-well plates at a density of 1×10^4^ cells per well, and treated with different concentrations of andrographolide for 24, 48 and 72 h. The cell viability was measured by CCK8 kit following manufacturer’s instructions. Similarly, non-small cell lung cancer (NSCLC) cells (5×10^3^ per well) were seeded into 96-well plates and treated with different concentrations of andrographolide for 24, 48 and 72 h. After treatment, cells were incubated with 500 μg/mL of MTT [3–(4,5-dimethyl-2-thiazolyl)-2,5-diphenyl-2-H-tetrazolium bromide] for 4 h, and the supernatants were removed. The plates were read at 490 nm using Synergy 2 Multi-Mode Microplate Reader (BioTek Instruments, Inc., Winooski, VT).

### Calcein Release Assay

NK cells cytotoxicity were determined by using the calcein release fluorometric assay [38, 39]. A549 cells were incubated with 2 μg/mL of calcein-AM at 37 °C for 1 h with occasional shaking. NK cells and A549 cells were mixed at effector-to-target (E:T) ratio of 5:1 (5×10^4^:1×10^4^) and co-cultured for 4 h. After incubation, 100 μL of supernatant was transferred to a new plate. The fluorescence of samples was measured with a Synergy 2 Multi-Mode Microplate Reader (BioTek Instruments, Inc., Winooski, VT) (excitation filter 485 nm, emission filter 538 nm). The percentage lysis was calculated according to the formula [(experimental release − spontaneous release)/(maximum release − spontaneous release)] × 100.

### Statistical Analysis

High-throughput screening assay was evaluated by Z’-factor as described previously [29, 30]. Results with Z’-factor below zero were discarded.

$$ \mathrm{Z}^{\prime }=1-\left(3*\mathrm{S}\mathrm{D}\mathrm{c}++3*\mathrm{S}\mathrm{D}\mathrm{c}-\right)/\left(\mu \mathrm{c}+-\mu \mathrm{c}-\right) $$

SDc+ standard deviation of IL-2 stimulated NK cell activity

SDc− standard deviation based on level of NK cell activity

μc+ mean of IL-2 stimulated NK cell activity

μc− mean based on level of NK cell activity

Results are expressed as the mean ± SEM. Statistical comparison in two groups was performed by Student’s *t*-test. *P*<0.05 represented statistical significant differences.
